# Improving the Antioxidant Properties of Tin-Based Perovskite for the Enhanced Performance of Near-Infrared Light-Emitting Diodes Through the Synergy of Sn and SnF_2_

**DOI:** 10.3390/ma17246059

**Published:** 2024-12-11

**Authors:** Yipeng Shen, Jianan Chen, Yuhan Si, Zhengguo Xiao, Kai Kang, Zhaobing Tang, Jing Wang, Chaoyu Xiang

**Affiliations:** 1 Laboratory of Optoelectronic and Information Materials and Devices, Ningbo Institute of Materials Technology and Engineering, Chinese Academy of Sciences, Ningbo 315201, China; qe77@mail.ustc.edu.cn (Y.S.); xiaonan19970905@163.com (J.C.); siyuhan@nimte.ac.cn (Y.S.); 2 Nano Science and Technology Institute, University of Science and Technology of China, Hefei 230026, China; zhengguo@ustc.edu.cn; 3 Laboratory of Optoelectronic and Information Materials and Devices, Qianwan Institute of CNITECH, Ningbo 315336, China; 4 Zhejiang Provincial Engineering Research Center of Energy Optoelectronic Materials and Devices, Ningbo Institute of Materials Technology and Engineering, Chinese Academy of Sciences, Ningbo 315201, China; 5 Department of Electrical and Electronic Engineering, University of Nottingham Ningbo China, Ningbo 315000, China; 6 The Institute of Advanced Displays and Imaging, Henan Academy of Sciences, Zhengzhou 450046, China; 7 Nottingham Ningbo China Beacons of Excellence Research and Innovation Institute, University of Nottingham Ningbo China, Ningbo 315048, China

**Keywords:** tin-based perovskite, light-emitting diodes, synergy, antioxidant

## Abstract

Tin-based perovskite has emerged as an excellent luminescent material due to its non-toxicity and narrow bandgap compared to lead-based perovskite. However, its tin ions are easily oxidized by oxygen, which leads to increased vacancy defects and poor crystallinity, presenting a significant challenge in obtaining high-quality perovskite films. In this context, we introduced an approach by synergistically adding SnF_2_ and tin powder into the precursor solution to enhance the antioxidation of Sn ions. This method effectively improved the crystallinity of the perovskite films, reduced the density of defect states, and enhanced the photoluminescence performance of the films. Based on these findings, we successfully fabricated tin-based near-infrared perovskite light-emitting diodes (PeLEDs). With a 20% improvement in the Sn^2+^ content in the film, we achieved a threefold increase in the external quantum efficiency of the devices, reaching 3.6%.

## 1. Introduction

Halide perovskites, as an excellent luminescent material, have been employed in the fabrication of near-infrared light-emitting diode (LED) devices with external quantum efficiencies surpassing 20% [1,2,3,4]. However, these accomplishments have predominantly stemmed from lead-based halide perovskites, which face challenges in meeting the increasingly stringent demands for environmental sustainability [5,6]. Tin-based perovskite, characterized by a radius similar than that of lead, has attracted substantial attention from researchers due to its low toxicity and narrow bandgap [7,8,9]. Nevertheless, the performance of tin-based perovskite LEDs significantly lags behind that of their lead-based counterparts. This could primarily be attributed to the absence of the lanthanide contraction effect in tin [10], resulting in a weaker attraction to the outermost electrons. This reduced affinity makes tin prone to losing two electrons from its 5s orbital, leading to a state of heightened chemical instability [11,12]. This easily leads to the oxidation of Sn^2+^ to Sn^4+^, resulting in the formation of vacancy defects and inducing significant non-radiative recombination losses, reducing the external quantum efficiency of the devices [13,14].

To address the issue of oxidation, various strategies have been employed [15,16]. Using inorganic reducing agents and tin compensation agents have been demonstrated as effective methods to prevent oxidation. Liang et al., for instance, introduced the inorganic acid H_3_PO_2_ to fabricate tin-based perovskite devices, effectively curtailing oxidation [17]. However, the employment of a water-based solvent, hypophosphorous acid, exhibited some negative impacts on the perovskite, posing challenges to further enhancements in the device performance. Meanwhile, Huang et al. successfully decreased the initial Sn^4+^ content within SnI_2_ by introducing tin powder into the FASnI_3_ precursor solution [18]. Research findings have indicated that the incorporation of tin compensation agents, including SnF_2_, SnCl_2_, SnBr_2_, and SnI_2_, within perovskite can effectively increase the chemical potential of tin, as well as the Sn vacancy formation energy [19,20]. Concurrently, the introduction of fluoride ions to replace iodine in SnI_4_ results in the formation of SnF_4_, effectively hindering the development of SnI_4_ within the film [21].

In this work, a collaborative approach involving the utilization of tin powder (an inorganic reducing agent) and the tin compensator SnF_2_ effectively decreased the Sn^4+^ content in both the precursor solution and the thin film. As a result, the Sn^4+^ content was effectively reduced by approximately 20%. Furthermore, the incorporation of SnF_2_ led to an improvement in the crystalline quality, manifested by a significant reduction in the defect density. This enhancement in crystalline quality was particularly attributed to the increase in the black orthorhombic phase (B-γ-CsSnI_3_) and the suppressing of the formation of the yellow phase (Y-CsSnI_3_). Building upon these effects, the device’s maximum external quantum efficiency underwent a threefold enhancement compared to its original performance level, with the highest EQE reaching 3.6%.

## 2. Materials and Methods

### 2.1. Material

The precursor solution was prepared by mixing CsI (Sigma-Aldrich, Darmstadt, Germany, 99.99%), SnI_2_ (99.999%), SnF_2_ (Macklin, Shanghai, China, 99.9%), and tin powder (Energy Chemical, Shanghai, China, 99.995%). N, N-dimethyl sulfoxide (DMSO, Sigma-Aldrich, Darmstadt, Germany, 99.9%), Poly(3,4-ethylene dioxythiophene): poly(styrene sulfonate) (PEDOT: PSS, Baolait Optoelectronic Technology, Xian, China, 4083), 4,6-bis(3,5-di-3-pyridylphenyl)-2-methylpyrimidine (B_3_PyMPM), lithium fluoride (LiF, 99.995%), and aluminum (Al, 99.999%) were used as received.

### 2.2. Preparation of Precursor Solution

To prepare a precursor solution with a concentration of 0.2 mmol/mL, CsI, SnI_2_, and SnF_2_ were mixed in a molar ratio of 1:1:0.1. Additionally, 5 mg of tin powder was dissolved in 1 mL of DMSO solvent, followed by stirring for 7 h.

### 2.3. Device Fabrication

The devices were fabricated on glass substrates coated with indium tin oxide (ITO). The substrates were sequentially cleaned by ultrasonication in deionized water, acetone, and isopropanol for 15 min each, followed by UV-ozone treatment for 15 min. A hole transport layer (HTL) was prepared on the ITO glass substrate by spin-coating PEDOT: PSS at 4000 rpm for 40 s. The coated layer was annealed at 150 °C for 15 min and then cooled to room temperature and transferred to an N_2_ glove box. For the emitting layer, 40 μL of the precursor solution was spin-coated onto the PEDOT: PSS layer at 5500 rpm for 60 s and annealed at 110 °C for 10 min. Lastly, under a vacuum pressure of <9 × 10^−5^ Pa, the electron transport layer B_3_PyMPM (ETL) with a thickness of 45 nm, a 1 nm-thick LiF layer, and a 100 nm-thick Al layer were thermally evaporated. The effective area defined by the overlap of the Al and ITO electrodes was 4 mm^2^.

### 2.4. Film Characterization

Calcium titanate thin films were examined using the HITACHI Regulus 8230 scanning electron microscope. X-ray diffraction (XRD) patterns were obtained with an X-ray diffractometer (Bruker D8, Advance) with a Cu Kα radiation source. The surface morphology of different layers was collected using an AFM (Bruker, Billerica, MA, USA, Dimension ICON). XPS spectra were acquired using an X-ray photoelectron spectroscopy instrument (AXIS SUPRA+). Steady-state photoluminescence (PL) spectra were obtained using in situ PL measurements. The photoluminescence quantum yield (PLQY) data were collected using an integrating sphere and a continuous 650 nm laser excitation source.

## 3. Results and Discussion

### 3.1. Environmental Stability of Perovskite Precursor Solution

**Figure 1:**
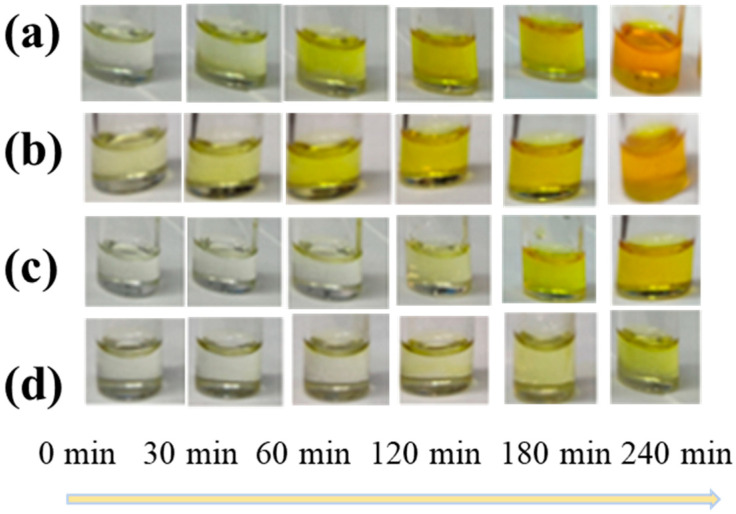
The color changes of CsSnI_3_ precursors with different additives upon oxidation in air (**a**) without any additive, (**b**) with only tin powder, (**c**) with 10% SnF_2_, (**d**) and with tin powder with 10% SnF_2_.

**Figure A1:**
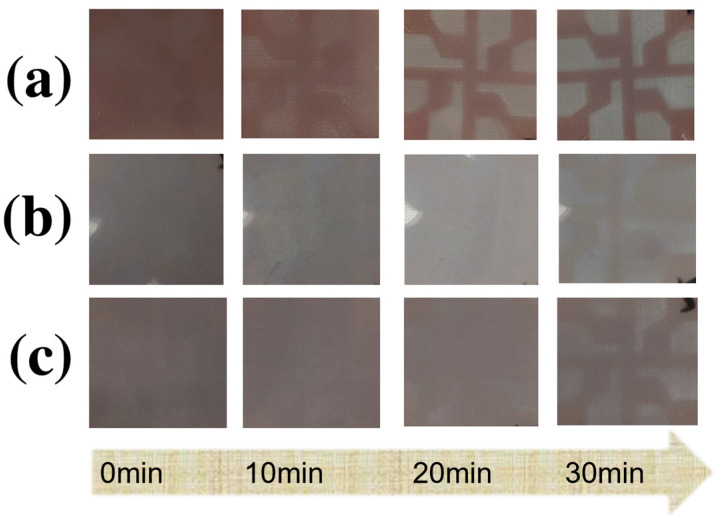
(**a**) Evolution of perovskite thin film without any additives in ambient air; (**b**) Evolution of perovskite thin film with only SnF_2_ added in ambient air; (**c**) Evolution of perovskite thin film with both SnF_2_ and tin powder added simultaneously.

First, we tested SnF_2_ and tin powder as reducing agents to mitigate the presence of Sn^4+^ ions in the precursor solution. The color changes in the precursor solutions exposed to air for different durations were demonstrated (Figure 1). Figure 1a,b correspond to scenarios where no additives were incorporated and only SnF_2_ was added. After 240 min, the solution transitioned from a light yellow to a deep brown color, primarily attributed to the oxidation of Sn^2+^ and the gradual increase in Sn^4+^ content. The color change noticeably slowed down when only tin powder was added (Figure 1c). However, the most effective antioxidation effect was observed when SnF_2_ and tin powder were added simultaneously (Figure 1d). Subsequently, we tested the air stability of the perovskite thin films treated with different additives, as depicted in Figure A1 (Appendix A). The pristine film showed slight degradation after 10 min, while the film treated with SnF_2_ exhibited relatively slower degradation. This indicates that SnF_2_ could enhance the stability of the film.

### 3.2. Analysis of Antioxidant Capacity of Tin-Based Perovskite Films

**Figure 2:**
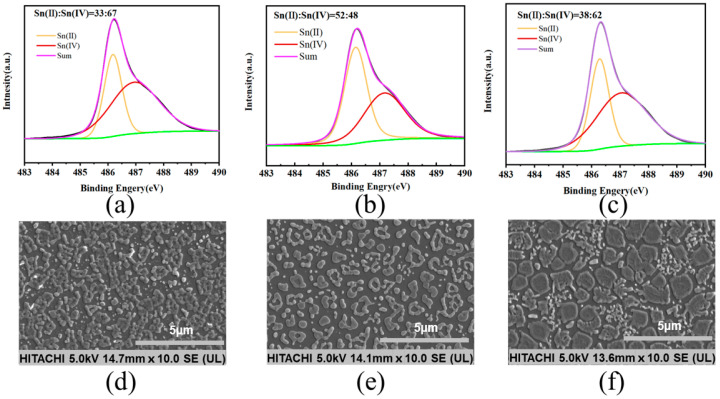
XPS spectra and SEM images of perovskite thin films with different additives: (**a**,**d**) without any additive, (**b**,**e**) with 10% SnF_2_ and tin powder, (**c**,**f**) and with only tin powder.

**Figure A2:**
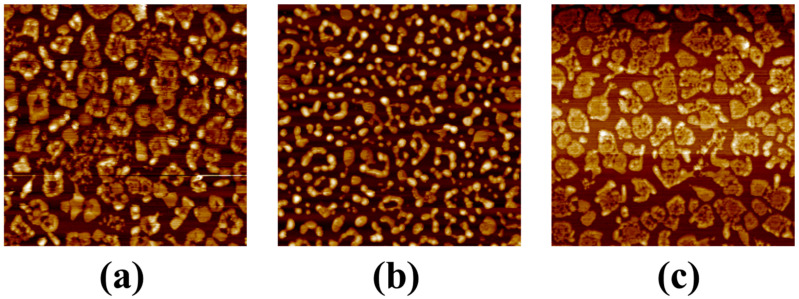
AFM images of perovskite thin films with different additives. (**a**) without any additive, (**b**) with 10% SnF_2_ and tin powder, (**c**) with only tin powder.

The role of SnF_2_ in inhibiting the oxidation of Sn^2+^ was revealed by X-ray photoelectron spectroscopy (XPS). The central peaks observed in the perovskite thin film at 486.3 eV and 487.4 eV corresponded to Sn^2+^ and Sn^4+^, respectively. As shown in Figure 2a, the original thin film exhibited a significantly higher proportion of Sn^4+^ compared to Sn^2+^, indicating that Sn^2+^ oxidized after the annealing process (Figure 2a). Upon introducing the reducing agent, tin powder, a slight increase in the Sn^2+^ content and a relative decrease in the Sn^4+^ content were observed in the thin film (Figure 2c). When SnF_2_ and tin powder were simultaneously added, the ratio of Sn^2+^ to Sn^4+^ increased from 33:67 in the original thin film to 52:48 (Figure 2b). We conducted surface morphology studies of the perovskite thin films using scanning electron microscopy (SEM). The original perovskite thin film exhibited a higher concentration of aggregated crystalline entities (Figure 2d). When both SnF_2_ and tin powder were added simultaneously, the distribution of thin film grains appeared more uniform, presenting as island-like structures (Figure 2e). However, films with only tin powder showed uneven crystallization with numerous clusters, potentially leading to non-radiative recombination and thus affecting device performance (Figure 2f) [22]. We demonstrated through atomic force microscopy (AFM) testing that the perovskite film with both tin powder and SnF_2_ had the best crystallinity and uniformity, with no clustering observed (Figure A2) [23].

### 3.3. Crystallography and Optical Properties of Tin-Based Perovskite Films

**Figure 3:**
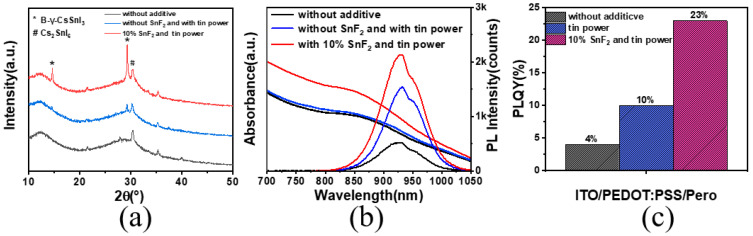
(**a**) XRD pattern with different additives. (**b**) Photoluminescence spectra and absorption spectra of perovskite. (**c**) Photoluminescence quantum yield values of perovskite thin films.

X-ray diffraction analysis was employed to investigate the influence of SnF_2_ on the crystallinity of the perovskite thin films (Figure 3a). The results revealed that the original films, without any additives, exhibited a minimal intensity around 14.5° and 29° in the diffraction spectrum, which corresponded to the (110) and (220) crystallographic planes of the black γ-CsSnI_3_ phase. When only tin powder was added, the corresponding peak intensities increased slightly. However, when both SnF_2_ and tin powder were added simultaneously, the intensity of the black phase peak significantly increased, indicating a more pronounced enhancement in crystallinity due to SnF_2_. We assessed the optical properties of the films to evaluate film quality, as shown in Figure 3b. The films with synergistic additives exhibited the highest photoluminescence intensity (PL) at 930 nm, along with a high absorption intensity. The films with both SnF_2_ and tin powder added showed an increased photoluminescence quantum yield (PLQY), five times higher than that of the original films. The enhancement of PLQY by adding only tin powder was not significant (Figure 3c).

### 3.4. Electrical Capability Analysis of Tin-Based Perovskite Devices

**Figure 4:**
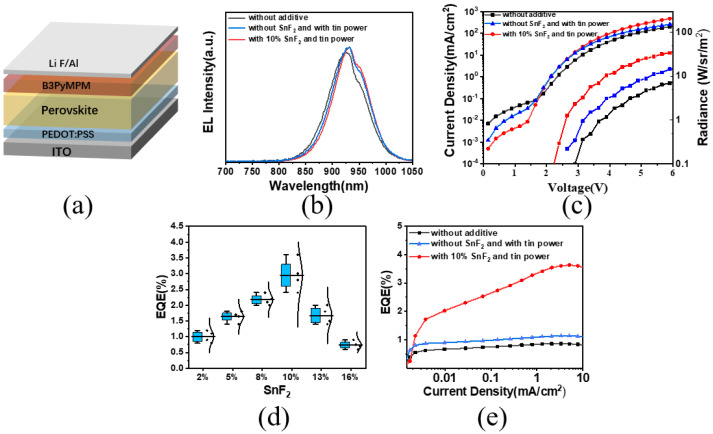
(**a**) LED device structure. (**b**) Electroluminescence spectra with different additives. (**c**) Current density and radiance of devices with different additives. (**d**) EQE values of devices with different concentrations of SnF_2_. (**e**) EQE values of devices with different additives.

**Figure A3:**
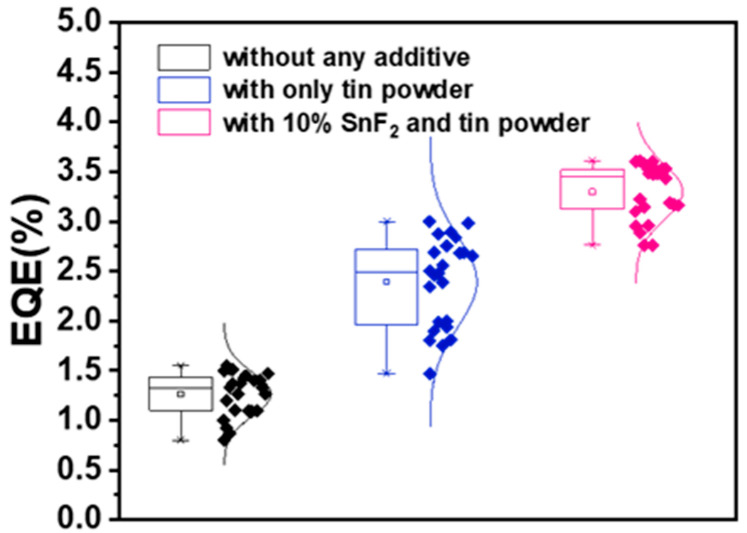
EQE statistics chart of tin-based perovskite devices with different additives.

**Figure A4:**
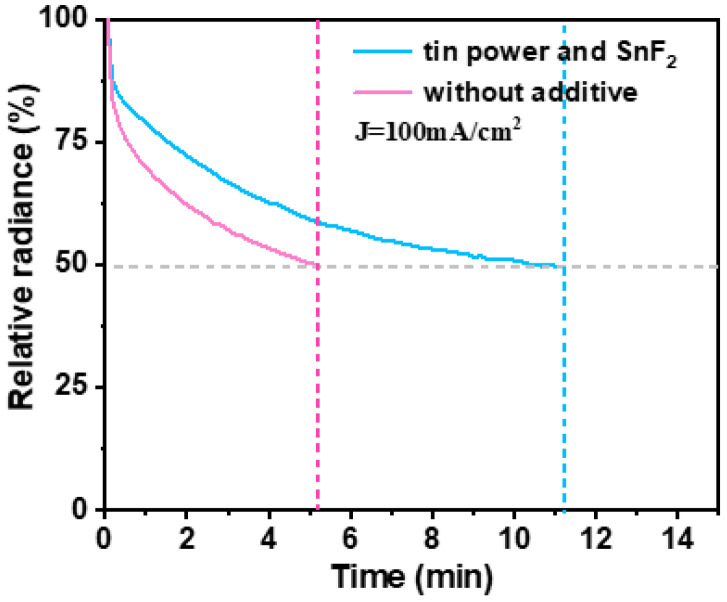
Device lifetime of tin-based perovskite devices in the air environment.

To investigate how additives influence device performance, PeLED devices were fabricated with a structure consisting of indium tin oxide (ITO)/Poly(3,4-ethylene dioxythiophene)-poly(styrene sulfonate) (PEDOT: PSS)/perovskite/4,6-bis(3,5-dipyridin-3-ylphenyl)-2-methylpyrimidine (B_3_PyMPM)/LiF/Al (Figure 4a). The additives did not alter the electroluminescence spectrum peak position of the devices (Figure 4b). Under a driving voltage of 4 V, devices with synergistic additives exhibited an increased current density and radiance compared to those from the original thin films and showed lower leakage current (Figure 4c). The cooperative action of SnF_2_ and tin powder resulted in an EQE of 3.6% for the devices, four times higher than the original devices (Figure 4e). We compiled the EQE data of tin-based perovskite devices with different additives and found that the simultaneous addition of tin powder and SnF_2_ significantly enhanced the devices’ EQE (Figure A3). Finally, we tested the lifetime of the tin-based perovskite devices, and the device with both tin powder and SnF_2_ additives had twice the lifetime of the device without additives (Figure A4). This enhancement was achieved by improving the crystallinity and uniformity of the thin films, and the synergistic effect of the additives led to a lower proportion of Sn^2+^ oxidation.

## 4. Conclusions

The synergistic effect of using SnF_2_ and tin powder significantly enhanced the performance of the devices. Characterization tests revealed that tin powder exhibited a pronounced antioxidation effect when introduced into the precursor solution. The simultaneous addition of SnF_2_ and tin powder effectively suppressed the oxidation of Sn^2+^ in the perovskite film, thereby reducing defects. Furthermore, the uniformity and crystallinity of the thin films showed notable improvements. Based on the improved antioxidation properties and enhanced film quality, the device efficiency demonstrated a substantial boost, with the highest EQE reaching 3.6%. These results underscore that enhancing the quality of perovskite thin films and mitigating the oxidation of tin ions by co-additives are effective strategies for enhancing the performance of tin-based near-infrared perovskite LEDs.

## Data Availability

The original contributions presented in this study are included in the article; further inquiries can be directed to the corresponding author.
